# Revolutionizing stroke therapy: microfluidic brain chips as a translational bridge

**DOI:** 10.1097/MS9.0000000000004337

**Published:** 2025-11-14

**Authors:** Muhammad Talha, Maliha Khalid, Aminath Waafira

**Affiliations:** a Department of Medicine, King Edward Medical University, Lahore, Pakistan; b Department of Medicine, Jinnah Sindh Medical University, Karachi, Pakistan; c Department of Medicine, The Maldives National University, Malé, Maldives

*To the Editor*,

Stroke continues to dominate among the leading causes of global mortality and disability, ischemic strokes constituting around 85% of all cases and a cost burden exceeding >72 billion dollars in the USA^[1]^. Existing therapies, such as thrombolysis and thrombectomy, have limited efficacy due to differences in clot composition among various patients, variations in how the neurovascular unit (NVU) functions with different types of strokes, and dissimilar mechanisms underlying post-stroke recovery^[2,3]^. Although microfluidic brain-on-chip (BoC) technology presents a promising platform for testing patient-specific therapies and modeling stroke pathophysiology, major technical and regulatory obstacles, such as high costs, the requirement for specialized labs, and limited scalability, are impeding its clinical translation^[1–3]^. This letter to the editor discusses the role of these chips in revolutionizing stroke therapy.

With structured building blocks that evoke human NVU functionality, microfluidic BoC devices present a dynamic control of the mock microenvironments for the interaction of brain microvascular endothelial cells, pericytes, astrocytes, neurons, and microglia from 3D co-cultures, facilitating the nutrient and oxygen exchange requirements. According to Lyu *et al*, the NVU-on-a-chip in their experiment mimicked the dynamics of the ischemic penumbra, where they showed that stem cell therapeutic interventions favored the restoration of blood–brain barrier integrity and preserved synaptic activity through modulation of the host cell interactions instead of involving direct replacement of neurons^[4]^. Within the framework of this platform, mesenchymal stem cells were able to achieve a 45% reduction in thrombus surface area under simulated flow conditions of the middle cerebral artery, indicating dose-dependent thrombolytic potential^[2]^. These observations accentuate the ability of BoC systems to more accurately assess the kinetics of drug delivery, clot lysis efficacy, and neurorestorative pathways with human-derived cells, further than animal studies, which mostly fail to reflect species-specific responses of the NVU^[1,5]^. BoC-derived barrier permeability and inflammatory responses have been linked to human stroke pathology in recent validation efforts; however, their reliability for clinical translation is limited by limited immune cell integration and long-term cellular behavior discrepancies^[6]^.

**HIGHLIGHTS**

- Stroke remains a leading global cause of mortality and disability, with current therapies limited by patient-specific variability.
- Microfluidic brain-on-chip (BoC) platforms replicate the neurovascular unit, enabling precise modeling of stroke pathophysiology and therapeutic responses.
- BoC systems hold promise for personalized stroke therapy and neurosurgical planning, although challenges in standardization and clinical translation remain.

Personalized stroke therapy comprises identifying patient-specific clot composition and the vulnerability of the NVU. A study in 2023 using a microfluidic model of thrombotic occlusion showed that the effectiveness of alteplase depends significantly on both thrombus retraction time (15% vs. 45% reperfusion at 0 vs. 6 hours post-occlusion) and dose^[2]^. Pre-testing of tetracycline agents/mechanical interventions in these setups with patient-derived endothelial cells and thrombi will optimize the reperfusion strategies. Moreover, BoC platforms incorporated with the brain extracellular matrix have been shown to enhance neuronal maturation and hypoxia response gene expression, thus paving the way for the tests of neuro-regenerative therapy^[5]^. These include other neurodegenerative diseases like Alzheimer’s, for example, modeling amyloid-beta aggregation and BBB disruption, and Parkinson’s, for example, studying dopaminergic neuron loss with alpha-synuclein pathology. Their versatility for neurological research is thus highlighted^[7]^. Despite these developments, BoC models still have significant drawbacks that prevent them from accurately simulating the neurovascular environment. These include poor vascular shear simulation, insufficient immune cell dynamics, and difficulties with long-term culture maintenance. Further impeding scalability and reproducibility are high manufacturing costs, lack of integration of high-throughput screening, and interlaboratory variability in chip design^[1,4]^.

BoC technology’s translational potential is still in its infancy. Despite their potential, human iPSC-derived BoC systems are not yet ready for clinical trials due to unresolved technical issues like standardizing chip designs and integrating complete immune responses. By co-culturing microglia or perfusing peripheral immune cells, for example, current models can partially replicate post-stroke inflammation; however, they are unable to fully capture the intricacy of immune dynamics during stroke recovery^[4,8,9]^. Although computational difficulties in data fusion still exist, researchers are tackling these problems by integrating multi-omics data (e.g., single-cell transcriptomics of thrombi on-chip) to improve biomarker identification and therapeutic targeting^[10]^. Significant obstacles also arise from ethical issues, such as data privacy in customized models, informed consent for the use of human-derived cells, and fair access to BoC technology^[8,9]^. Phase II clinical trial timelines of 5–10 years highlight the immaturity of existing BoC applications, requiring a cautious and balanced view of their clinical utility^[6,10]^.

Collaborative validation frameworks are critical to the advancement of BoC technology. Evidence must be consolidated through systematic reviews and meta-analyses, but the field needs more reliable, standardized datasets to guarantee methodological rigor^[10,11]^. In line with the TITAN guidelines for transparency in AI-driven healthcare, ongoing NIH-funded research attempts to create automated, clinical-grade BoC platforms for preoperative planning and post-stroke rehabilitation^[12]^. However, the revolutionary potential of BoC technology will remain restricted to preclinical research in the near future unless urgent issues like increasing cellular diversity, cutting costs, and establishing regulatory standards are resolved.

This microfluidic BoC technology has revolutionized therapy development in stroke disorders. Such systems overcome this difference in *in vitro* models versus human physiologies, enabling customized reperfusion and neurorestorative strategies. These efforts will continue to gain momentum while keeping the main NIH-funded research thrusts focused on the development of automated, clinical-grade BoC platforms for preoperative planning and rehabilitation post stroke. Our work is in line with the TITAN guidelines on the need for transparency in AI use in healthcare^[12]^.

## Data Availability

No data were generated for this manuscript.

## Research registration unique identifying number (UIN)

1. Name of the registry: Not applicable. 2. Unique identifying number or registration ID: Not applicable. 3. Hyperlink to your specific registration (must be publicly accessible and will be checked): Not applicable.
